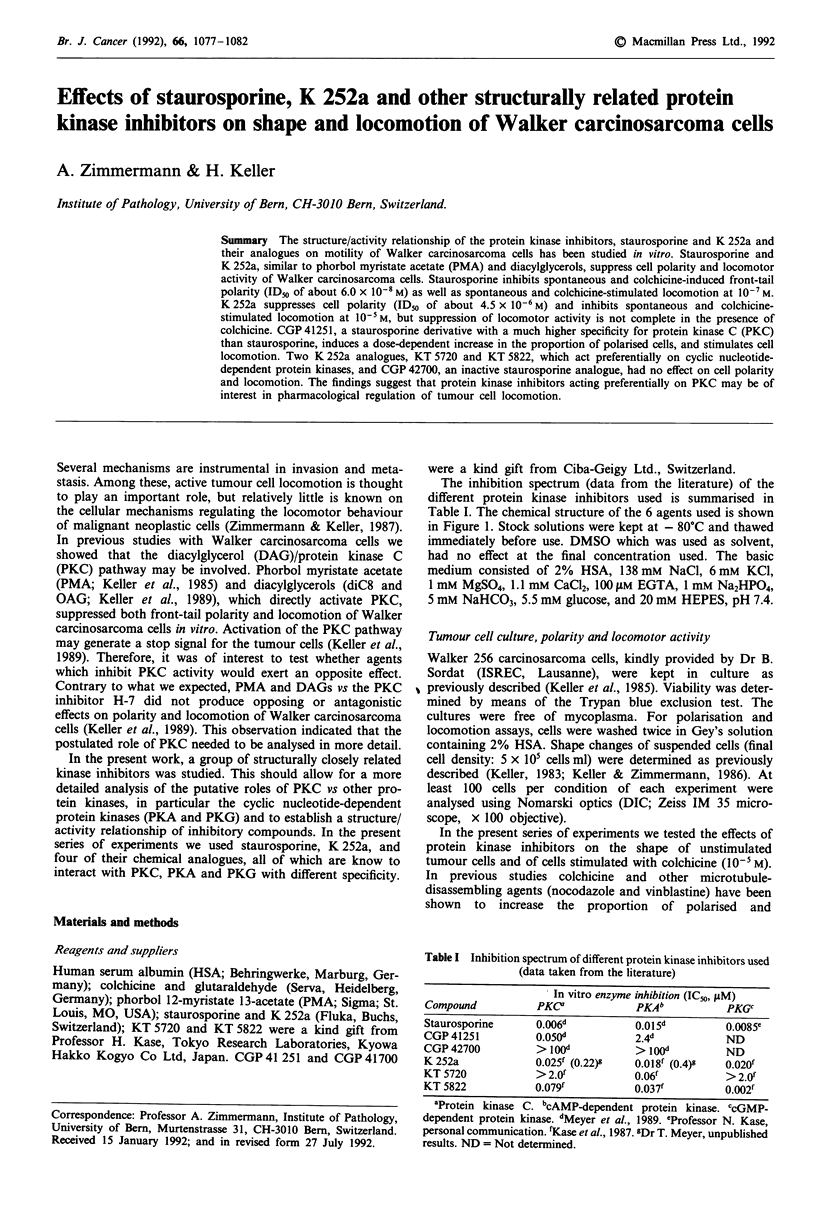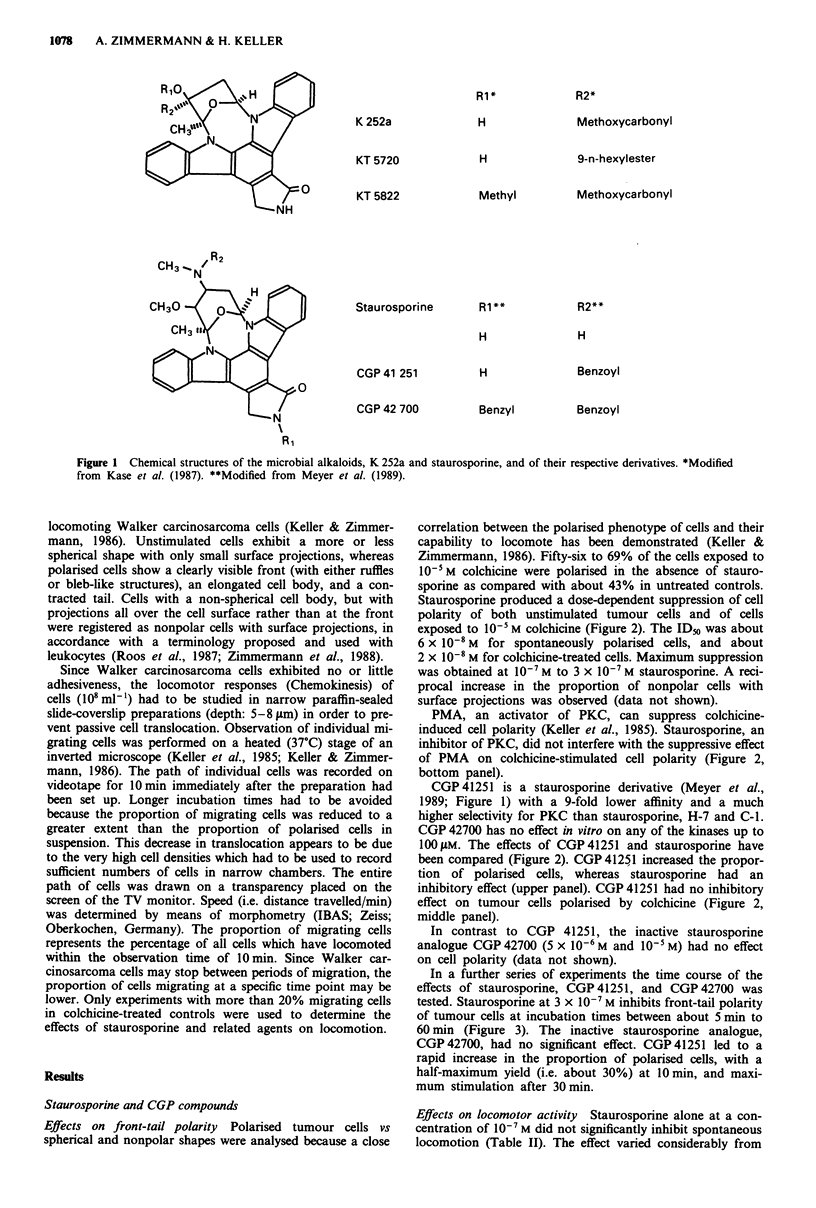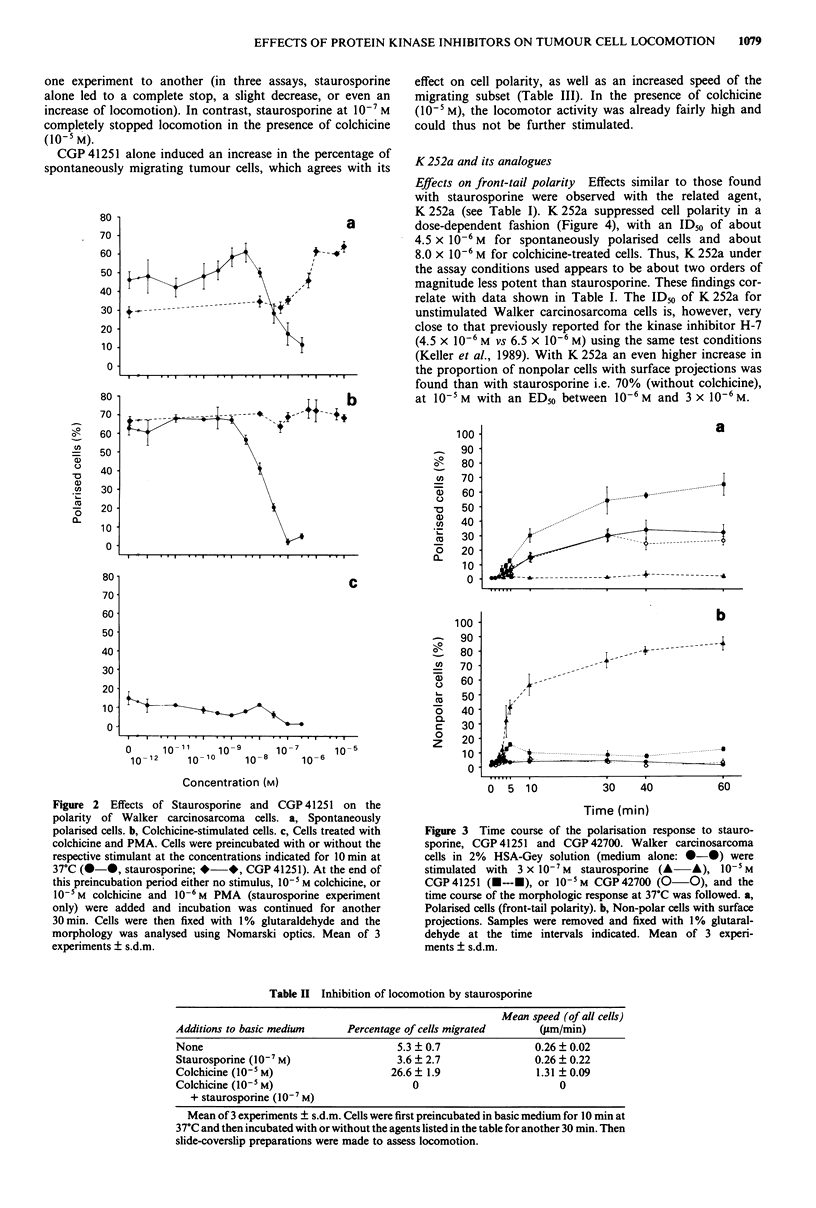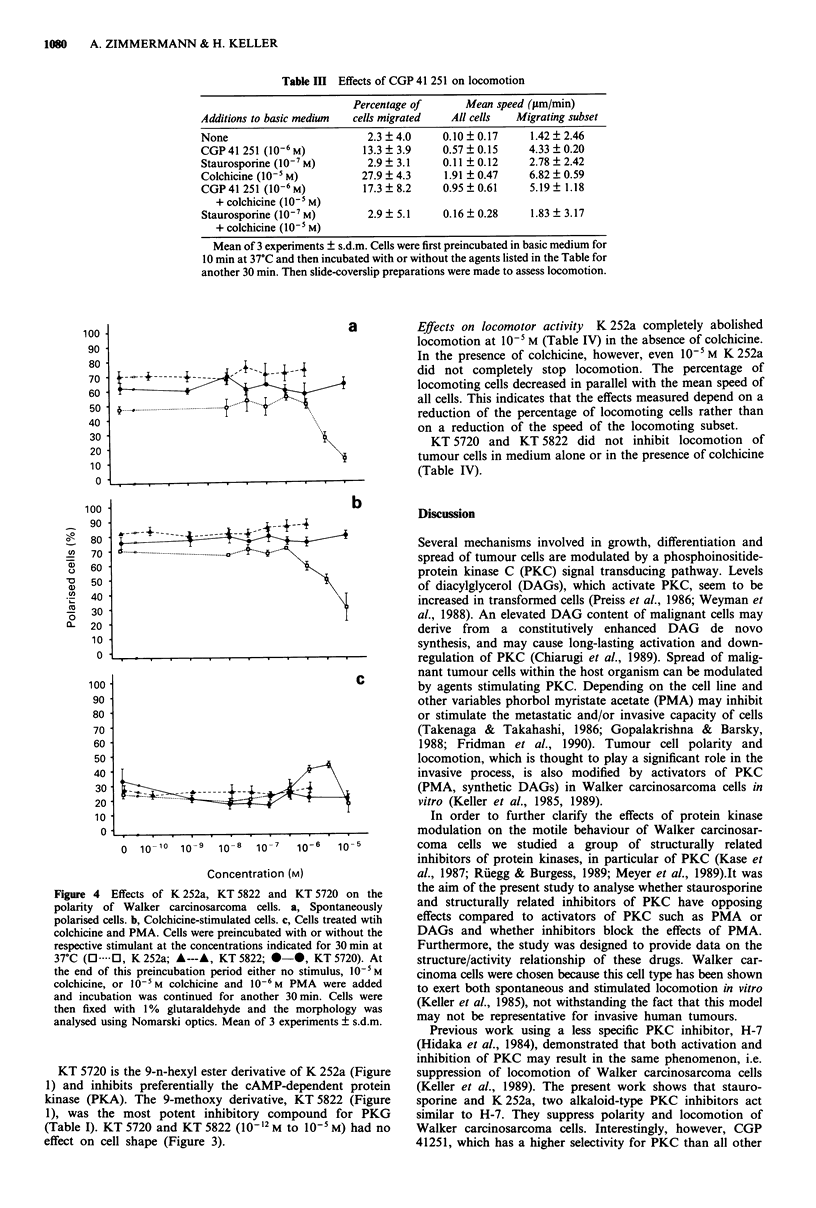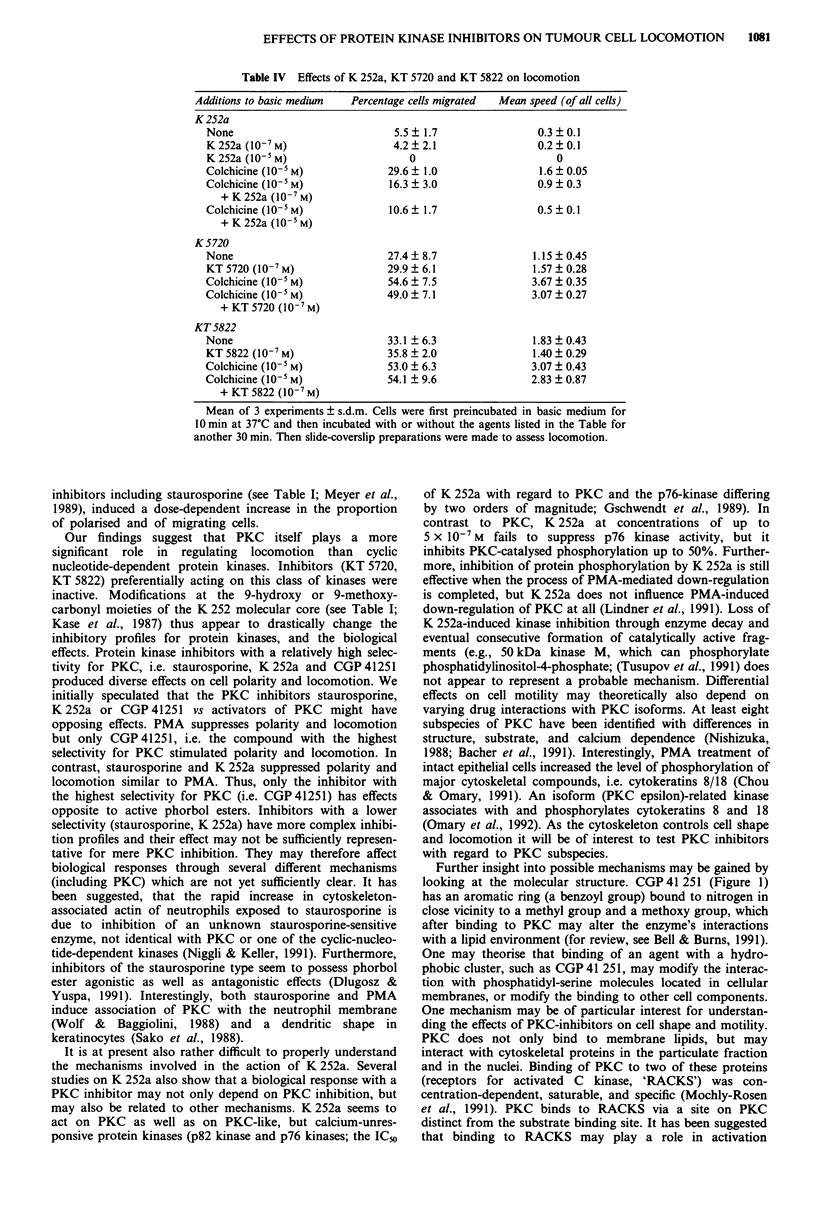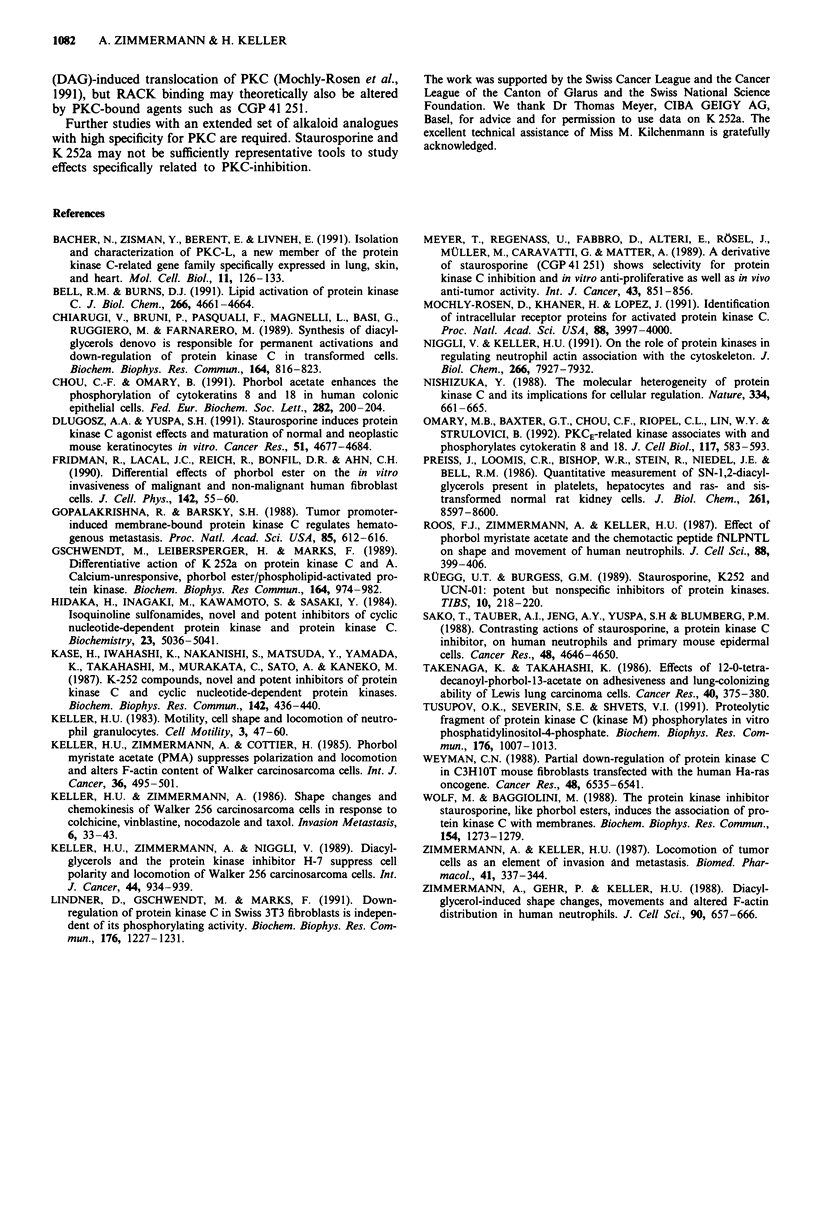# Effects of staurosporine, K 252a and other structurally related protein kinase inhibitors on shape and locomotion of Walker carcinosarcoma cells.

**DOI:** 10.1038/bjc.1992.413

**Published:** 1992-12

**Authors:** A. Zimmermann, H. Keller

**Affiliations:** Institute of Pathology, University of Bern, Switzerland.

## Abstract

The structure/activity relationship of the protein kinase inhibitors, staurosporine and K 252a and their analogues on motility of Walker carcinosarcoma cells has been studied in vitro. Staurosporine and K 252a, similar to phorbol myristate acetate (PMA) and diacylglycerols, suppress cell polarity and locomotor activity of Walker carcinosarcoma cells. Staurosporine inhibits spontaneous and colchicine-induced front-tail polarity (ID50 of about 6.0 x 10(-8) M) as well as spontaneous and colchicine-stimulated locomotion at 10(-7) M. K 252a suppresses cell polarity (ID50 of about 4.5 x 10(-6) M) and inhibits spontaneous and colchicine-stimulated locomotion at 10(-5) M, but suppression of locomotor activity is not complete in the presence of colchicine. CGP 41251, a staurosporine derivative with a much higher specificity for protein kinase C (PKC) than staurosporine, induces a dose-dependent increase in the proportion of polarised cells, and stimulates cell locomotion. Two K252a analogues, KT 5720 and KT 5822, which act preferentially on cyclic nucleotide-dependent protein kinases, and CGP 42700, an inactive staurosporine analogue, had no effect on cell polarity and locomotion. The findings suggest that protein kinase inhibitors acting preferentially on PKC may be of interest in pharmacological regulation of tumour cell locomotion.


*Br. J. Cancer* (1992), **66**, 1077–1082 © Macmillan Press Ltd., 1992

### Effects of staurosporine, K 252a and other structurally related protein kinase inhibitors on shape and locomotion of Walker carcinosarcoma cells

A. Zimmermann & H. Keller

*Institute of Pathology, University of Bern, CH-3010 Bern, Switzerland.*

**Summary** The structure/activity relationship of the protein kinase inhibitors, staurosporine and K 252a and their analogues on motility of Walker carcinosarcoma cells has been studied *in vitro*. Staurosporine and K 252a, similar to phorbol myristate acetate (PMA) and diacylglycerols, suppress cell polarity and locomotor activity of Walker carcinosarcoma cells. Staurosporine inhibits spontaneous and colchicine-induced front-tail polarity ($ID_{50}$ of about $6.0 \times 10^{-8}$ M) as well as spontaneous and colchicine-stimulated locomotion at $10^{-7}$ M. K 252a suppresses cell polarity ($ID_{50}$ of about $4.5 \times 10^{-6}$ M) and inhibits spontaneous and colchicine-stimulated locomotion at $10^{-5}$ M, but suppression of locomotor activity is not complete in the presence of colchicine. CGP 41251, a staurosporine derivative with a much higher specificity for protein kinase C (PKC) than staurosporine, induces a dose-dependent increase in the proportion of polarised cells, and stimulates cell locomotion. Two K 252a analogues, KT 5720 and KT 5822, which act preferentially on cyclic nucleotide-dependent protein kinases, and CGP 42700, an inactive staurosporine analogue, had no effect on cell polarity and locomotion. The findings suggest that protein kinase inhibitors acting preferentially on PKC may be of interest in pharmacological regulation of tumour cell locomotion.

Several mechanisms are instrumental in invasion and metastasis. Among these, active tumour cell locomotion is thought to play an important role, but relatively little is known on the cellular mechanisms regulating the locomotor behaviour of malignant neoplastic cells (Zimmermann & Keller, 1987). In previous studies with Walker carcinosarcoma cells we showed that the diacylglycerol (DAG)/protein kinase C (PKC) pathway may be involved. Phorbol myristate acetate (PMA; Keller *et al.*, 1985) and diacylglycerols (diC8 and OAG; Keller *et al.*, 1989), which directly activate PKC, suppressed both front-tail polarity and locomotion of Walker carcinosarcoma cells *in vitro*. Activation of the PKC pathway may generate a stop signal for the tumour cells (Keller *et al.*, 1989). Therefore, it was of interest to test whether agents which inhibit PKC activity would exert an opposite effect. Contrary to what we expected, PMA and DAGs *vs* the PKC inhibitor H-7 did not produce opposing or antagonistic effects on polarity and locomotion of Walker carcinosarcoma cells (Keller *et al.*, 1989). This observation indicated that the postulated role of PKC needed to be analysed in more detail.

In the present work, a group of structurally closely related kinase inhibitors was studied. This should allow for a more detailed analysis of the putative roles of PKC *vs* other protein kinases, in particular the cyclic nucleotide-dependent protein kinases (PKA and PKG) and to establish a structure/activity relationship of inhibitory compounds. In the present series of experiments we used staurosporine, K 252a, and four of their chemical analogues, all of which are know to interact with PKC, PKA and PKG with different specificity.

### Materials and methods

### *Reagents and suppliers*

Human serum albumin (HSA; Behringwerke, Marburg, Germany); colchicine and glutaraldehyde (Serva, Heidelberg, Germany); phorbol 12-myristate 13-acetate (PMA; Sigma; St. Louis, MO, USA); staurosporine and K 252a (Fluka, Buchs, Switzerland); KT 5720 and KT 5822 were a kind gift from Professor H. Kase, Tokyo Research Laboratories, Kyowa Hakko Kogyo Co Ltd, Japan. CGP 41 251 and CGP 41700 were a kind gift from Ciba-Geigy Ltd., Switzerland.

The inhibition spectrum (data from the literature) of the different protein kinase inhibitors used is summarised in Table I. The chemical structure of the 6 agents used is shown in Figure 1. Stock solutions were kept at − 80°C and thawed immediately before use. DMSO which was used as solvent, had no effect at the final concentration used. The basic medium consisted of 2% HSA, 138 mM NaCl, 6 mM KCl, 1 mM $MgSO_4$, 1.1 mM $CaCl_2$, 100 µM EGTA, 1 mM $Na_2HPO_4$, 5 mM $NaHCO_3$, 5.5 mM glucose, and 20 mM HEPES, pH 7.4.

### *Tumour cell culture, polarity and locomotor activity*

Walker 256 carcinosarcoma cells, kindly provided by Dr B. Sordat (ISREC, Lausanne), were kept in culture as previously described (Keller *et al.*, 1985). Viability was determined by means of the Trypan blue exclusion test. The cultures were free of mycoplasma. For polarisation and locomotion assays, cells were washed twice in Gey's solution containing 2% HSA. Shape changes of suspended cells (final cell density: $5 \times 10^5$ cells ml) were determined as previously described (Keller, 1983; Keller & Zimmermann, 1986). At least 100 cells per condition of each experiment were analysed using Nomarski optics (DIC; Zeiss IM 35 microscope, × 100 objective).

In the present series of experiments we tested the effects of protein kinase inhibitors on the shape of unstimulated tumour cells and of cells stimulated with colchicine ($10^{-5}$ M). In previous studies colchicine and other microtubule-disassembling agents (nocodazole and vinblastine) have been shown to increase the proportion of polarised and

**Table I** Inhibition spectrum of different protein kinase inhibitors used (data taken from the literature)

| *Compound* | In vitro *enzyme inhibition* ($IC_{50}$, µM) *PKC*[a] | *PKA*[b] | *PKG*[c] |
|---|---|---|---|
| Staurosporine | 0.006[d] | 0.015[d] | 0.0085[e] |
| CGP 41251 | 0.050[d] | 2.4[d] | ND |
| CGP 42700 | >100[d] | >100[d] | ND |
| K 252a | 0.025[f] (0.22)[g] | 0.018[f] (0.4)[g] | 0.020[f] |
| KT 5720 | >2.0[f] | 0.06[f] | >2.0[f] |
| KT 5822 | 0.079[f] | 0.037[f] | 0.002[f] |

[a]Protein kinase C. [b]cAMP-dependent protein kinase. [c]cGMP-dependent protein kinase. [d]Meyer *et al.*, 1989. [e]Professor N. Kase, personal communication. [f]Kase *et al.*, 1987. [g]Dr T. Meyer, unpublished results. ND = Not determined.

Correspondence: Professor A. Zimmermann, Institute of Pathology, University of Bern, Murtenstrasse 31, CH-3010 Bern, Switzerland.
Received 15 January 1992; and in revised form 27 July 1992.

| | R1* | R2* |
|---|---|---|
| K 252a | H | Methoxycarbonyl |
| KT 5720 | H | 9-n-hexylester |
| KT 5822 | Methyl | Methoxycarbonyl |

| Staurosporine | R1** | R2** |
|---|---|---|
| | H | H |
| CGP 41 251 | H | Benzoyl |
| CGP 42 700 | Benzyl | Benzoyl |

**Figure 1** Chemical structures of the microbial alkaloids, K 252a and staurosporine, and of their respective derivatives. *Modified from Kase *et al.* (1987). **Modified from Meyer *et al.* (1989).

locomoting Walker carcinosarcoma cells (Keller & Zimmermann, 1986). Unstimulated cells exhibit a more or less spherical shape with only small surface projections, whereas polarised cells show a clearly visible front (with either ruffles or bleb-like structures), an elongated cell body, and a contracted tail. Cells with a non-spherical cell body, but with projections all over the cell surface rather than at the front were registered as nonpolar cells with surface projections, in accordance with a terminology proposed and used with leukocytes (Roos *et al.*, 1987; Zimmermann *et al.*, 1988).

Since Walker carcinosarcoma cells exhibited no or little adhesiveness, the locomotor responses (Chemokinesis) of cells ($10^8$ ml$^{-1}$) had to be studied in narrow paraffin-sealed slide-coverslip preparations (depth: 5–8 μm) in order to prevent passive cell translocation. Observation of individual migrating cells was performed on a heated (37°C) stage of an inverted microscope (Keller *et al.*, 1985; Keller & Zimmermann, 1986). The path of individual cells was recorded on videotape for 10 min immediately after the preparation had been set up. Longer incubation times had to be avoided because the proportion of migrating cells was reduced to a greater extent than the proportion of polarised cells in suspension. This decrease in translocation appears to be due to the very high cell densities which had to be used to record sufficient numbers of cells in narrow chambers. The entire path of cells was drawn on a transparency placed on the screen of the TV monitor. Speed (i.e. distance travelled/min) was determined by means of morphometry (IBAS; Zeiss; Oberkochen, Germany). The proportion of migrating cells represents the percentage of all cells which have locomoted within the observation time of 10 min. Since Walker carcinosarcoma cells may stop between periods of migration, the proportion of cells migrating at a specific time point may be lower. Only experiments with more than 20% migrating cells in colchicine-treated controls were used to determine the effects of staurosporine and related agents on locomotion.

### Results

### *Staurosporine and CGP compounds*

*Effects on front-tail polarity* Polarised tumour cells *vs* spherical and nonpolar shapes were analysed because a close correlation between the polarised phenotype of cells and their capability to locomote has been demonstrated (Keller & Zimmermann, 1986). Fifty-six to 69% of the cells exposed to $10^{-5}$ M colchicine were polarised in the absence of staurosporine as compared with about 43% in untreated controls. Staurosporine produced a dose-dependent suppression of cell polarity of both unstimulated tumour cells and of cells exposed to $10^{-5}$ M colchicine (Figure 2). The $ID_{50}$ was about $6 \times 10^{-8}$ M for spontaneously polarised cells, and about $2 \times 10^{-8}$ M for colchicine-treated cells. Maximum suppression was obtained at $10^{-7}$ M to $3 \times 10^{-7}$ M staurosporine. A reciprocal increase in the proportion of nonpolar cells with surface projections was observed (data not shown).

PMA, an activator of PKC, can suppress colchicine-induced cell polarity (Keller *et al.*, 1985). Staurosporine, an inhibitor of PKC, did not interfere with the suppressive effect of PMA on colchicine-stimulated cell polarity (Figure 2, bottom panel).

CGP 41251 is a staurosporine derivative (Meyer *et al.*, 1989; Figure 1) with a 9-fold lower affinity and a much higher selectivity for PKC than staurosporine, H-7 and C-1. CGP 42700 has no effect *in vitro* on any of the kinases up to 100 μM. The effects of CGP 41251 and staurosporine have been compared (Figure 2). CGP 41251 increased the proportion of polarised cells, whereas staurosporine had an inhibitory effect (upper panel). CGP 41251 had no inhibitory effect on tumour cells polarised by colchicine (Figure 2, middle panel).

In contrast to CGP 41251, the inactive staurosporine analogue CGP 42700 ($5 \times 10^{-6}$ M and $10^{-5}$ M) had no effect on cell polarity (data not shown).

In a further series of experiments the time course of the effects of staurosporine, CGP 41251, and CGP 42700 was tested. Staurosporine at $3 \times 10^{-7}$ M inhibits front-tail polarity of tumour cells at incubation times between about 5 min to 60 min (Figure 3). The inactive staurosporine analogue, CGP 42700, had no significant effect. CGP 41251 led to a rapid increase in the proportion of polarised cells, with a half-maximum yield (i.e. about 30%) at 10 min, and maximum stimulation after 30 min.

*Effects on locomotor activity* Staurosporine alone at a concentration of $10^{-7}$ M did not significantly inhibit spontaneous locomotion (Table II). The effect varied considerably from one experiment to another (in three assays, staurosporine alone led to a complete stop, a slight decrease, or even an increase of locomotion). In contrast, staurosporine at $10^{-7}$ M completely stopped locomotion in the presence of colchicine ($10^{-5}$ M).

CGP 41251 alone induced an increase in the percentage of spontaneously migrating tumour cells, which agrees with its effect on cell polarity, as well as an increased speed of the migrating subset (Table III). In the presence of colchicine ($10^{-5}$ M), the locomotor activity was already fairly high and could thus not be further stimulated.

### *K 252a and its analogues*

*Effects on front-tail polarity* Effects similar to those found with staurosporine were observed with the related agent, K 252a (see Table I). K 252a suppressed cell polarity in a dose-dependent fashion (Figure 4), with an $ID_{50}$ of about $4.5 \times 10^{-6}$ M for spontaneously polarised cells and about $8.0 \times 10^{-6}$ M for colchicine-treated cells. Thus, K 252a under the assay conditions used appears to be about two orders of magnitude less potent than staurosporine. These findings correlate with data shown in Table I. The $ID_{50}$ of K 252a for unstimulated Walker carcinosarcoma cells is, however, very close to that previously reported for the kinase inhibitor H-7 ($4.5 \times 10^{-6}$ M *vs* $6.5 \times 10^{-6}$ M) using the same test conditions (Keller *et al.*, 1989). With K 252a an even higher increase in the proportion of nonpolar cells with surface projections was found than with staurosporine i.e. 70% (without colchicine), at $10^{-5}$ M with an $ED_{50}$ between $10^{-6}$ M and $3 \times 10^{-6}$ M.

**Figure 2** Effects of Staurosporine and CGP 41251 on the polarity of Walker carcinosarcoma cells. **a**, Spontaneously polarised cells. **b**, Colchicine-stimulated cells. **c**, Cells treated with colchicine and PMA. Cells were preincubated with or without the respective stimulant at the concentrations indicated for 10 min at 37°C (●—●, staurosporine; ◆——◆, CGP 41251). At the end of this preincubation period either no stimulus, $10^{-5}$ M colchicine, or $10^{-5}$ M colchicine and $10^{-6}$ M PMA (staurosporine experiment only) were added and incubation was continued for another 30 min. Cells were then fixed with 1% glutaraldehyde and the morphology was analysed using Nomarski optics. Mean of 3 experiments ± s.d.m.

**Figure 3** Time course of the polarisation response to staurosporine, CGP 41251 and CGP 42700. Walker carcinosarcoma cells in 2% HSA-Gey solution (medium alone: ●—●) were stimulated with $3 \times 10^{-7}$ M staurosporine (▲——▲), $10^{-5}$ M CGP 41251 (■---■), or $10^{-5}$ M CGP 42700 (O——O), and the time course of the morphologic response at 37°C was followed. **a**, Polarised cells (front-tail polarity). **b**, Non-polar cells with surface projections. Samples were removed and fixed with 1% glutaraldehyde at the time intervals indicated. Mean of 3 experiments ± s.d.m.

**Table II** Inhibition of locomotion by staurosporine

| *Additions to basic medium* | *Percentage of cells migrated* | *Mean speed (of all cells)* (μm/min) |
|---|---|---|
| None | 5.3 ± 0.7 | 0.26 ± 0.02 |
| Staurosporine ($10^{-7}$ M) | 3.6 ± 2.7 | 0.26 ± 0.22 |
| Colchicine ($10^{-5}$ M) | 26.6 ± 1.9 | 1.31 ± 0.09 |
| Colchicine ($10^{-5}$ M) + staurosporine ($10^{-7}$ M) | 0 | 0 |

Mean of 3 experiments ± s.d.m. Cells were first preincubated in basic medium for 10 min at 37°C and then incubated with or without the agents listed in the table for another 30 min. Then slide-coverslip preparations were made to assess locomotion.

**Table III** Effects of CGP 41 251 on locomotion

| *Additions to basic medium* | *Percentage of cells migrated* | *Mean speed (μm/min) All cells* | *Migrating subset* |
|---|---|---|---|
| None | 2.3 ± 4.0 | 0.10 ± 0.17 | 1.42 ± 2.46 |
| CGP 41 251 ($10^{-6}$ M) | 13.3 ± 3.9 | 0.57 ± 0.15 | 4.33 ± 0.20 |
| Staurosporine ($10^{-7}$ M) | 2.9 ± 3.1 | 0.11 ± 0.12 | 2.78 ± 2.42 |
| Colchicine ($10^{-5}$ M) | 27.9 ± 4.3 | 1.91 ± 0.47 | 6.82 ± 0.59 |
| CGP 41 251 ($10^{-6}$ M) + colchicine ($10^{-5}$ M) | 17.3 ± 8.2 | 0.95 ± 0.61 | 5.19 ± 1.18 |
| Staurosporine ($10^{-7}$ M) + colchicine ($10^{-5}$ M) | 2.9 ± 5.1 | 0.16 ± 0.28 | 1.83 ± 3.17 |

Mean of 3 experiments ± s.d.m. Cells were first preincubated in basic medium for 10 min at 37°C and then incubated with or without the agents listed in the Table for another 30 min. Then slide-coverslip preparations were made to assess locomotion.

**Figure 4** Effects of K 252a, KT 5822 and KT 5720 on the polarity of Walker carcinosarcoma cells. **a**, Spontaneously polarised cells. **b**, Colchicine-stimulated cells. **c**, Cells treated wtih colchicine and PMA. Cells were preincubated with or without the respective stimulant at the concentrations indicated for 30 min at 37°C (□····□, K 252a; ▲---▲, KT 5822; ●—●, KT 5720). At the end of this preincubation period either no stimulus, $10^{-5}$ M colchicine, or $10^{-5}$ M colchicine and $10^{-6}$ M PMA were added and incubation was continued for another 30 min. Cells were then fixed with 1% glutaraldehyde and the morphology was analysed using Nomarski optics. Mean of 3 experiments ± s.d.m.

KT 5720 is the 9-n-hexyl ester derivative of K 252a (Figure 1) and inhibits preferentially the cAMP-dependent protein kinase (PKA). The 9-methoxy derivative, KT 5822 (Figure 1), was the most potent inhibitory compound for PKG (Table I). KT 5720 and KT 5822 ($10^{-12}$ M to $10^{-5}$ M) had no effect on cell shape (Figure 3).

*Effects on locomotor activity* K 252a completely abolished locomotion at $10^{-5}$ M (Table IV) in the absence of colchicine. In the presence of colchicine, however, even $10^{-5}$ M K 252a did not completely stop locomotion. The percentage of locomoting cells decreased in parallel with the mean speed of all cells. This indicates that the effects measured depend on a reduction of the percentage of locomoting cells rather than on a reduction of the speed of the locomoting subset.

KT 5720 and KT 5822 did not inhibit locomotion of tumour cells in medium alone or in the presence of colchicine (Table IV).

### Discussion

Several mechanisms involved in growth, differentiation and spread of tumour cells are modulated by a phosphoinositide-protein kinase C (PKC) signal transducing pathway. Levels of diacylglycerol (DAGs), which activate PKC, seem to be increased in transformed cells (Preiss *et al.*, 1986; Weyman *et al.*, 1988). An elevated DAG content of malignant cells may derive from a constitutively enhanced DAG de novo synthesis, and may cause long-lasting activation and down-regulation of PKC (Chiarugi *et al.*, 1989). Spread of malignant tumour cells within the host organism can be modulated by agents stimulating PKC. Depending on the cell line and other variables phorbol myristate acetate (PMA) may inhibit or stimulate the metastatic and/or invasive capacity of cells (Takenaga & Takahashi, 1986; Gopalakrishna & Barsky, 1988; Fridman *et al.*, 1990). Tumour cell polarity and locomotion, which is thought to play a significant role in the invasive process, is also modified by activators of PKC (PMA, synthetic DAGs) in Walker carcinosarcoma cells *in vitro* (Keller *et al.*, 1985, 1989).

In order to further clarify the effects of protein kinase modulation on the motile behaviour of Walker carcinosarcoma cells we studied a group of structurally related inhibitors of protein kinases, in particular of PKC (Kase *et al.*, 1987; Rüegg & Burgess, 1989; Meyer *et al.*, 1989).It was the aim of the present study to analyse whether staurosporine and structurally related inhibitors of PKC have opposing effects compared to activators of PKC such as PMA or DAGs and whether inhibitors block the effects of PMA. Furthermore, the study was designed to provide data on the structure/activity relationship of these drugs. Walker carcinoma cells were chosen because this cell type has been shown to exert both spontaneous and stimulated locomotion *in vitro* (Keller *et al.*, 1985), not withstanding the fact that this model may not be representative for invasive human tumours.

Previous work using a less specific PKC inhibitor, H-7 (Hidaka *et al.*, 1984), demonstrated that both activation and inhibition of PKC may result in the same phenomenon, i.e. suppression of locomotion of Walker carcinosarcoma cells (Keller *et al.*, 1989). The present work shows that staurosporine and K 252a, two alkaloid-type PKC inhibitors act similar to H-7. They suppress polarity and locomotion of Walker carcinosarcoma cells. Interestingly, however, CGP 41251, which has a higher selectivity for PKC than all other

**Table IV** Effects of K 252a, KT 5720 and KT 5822 on locomotion

| *Additions to basic medium* | *Percentage cells migrated* | *Mean speed (of all cells)* |
|---|---|---|
| *K 252a* | | |
| None | 5.5 ± 1.7 | 0.3 ± 0.1 |
| K 252a ($10^{-7}$ M) | 4.2 ± 2.1 | 0.2 ± 0.1 |
| K 252a ($10^{-5}$ M) | 0 | 0 |
| Colchicine ($10^{-5}$ M) | 29.6 ± 1.0 | 1.6 ± 0.05 |
| Colchicine ($10^{-5}$ M) + K 252a ($10^{-7}$ M) | 16.3 ± 3.0 | 0.9 ± 0.3 |
| Colchicine ($10^{-5}$ M) + K 252a ($10^{-5}$ M) | 10.6 ± 1.7 | 0.5 ± 0.1 |
| *K 5720* | | |
| None | 27.4 ± 8.7 | 1.15 ± 0.45 |
| KT 5720 ($10^{-7}$ M) | 29.9 ± 6.1 | 1.57 ± 0.28 |
| Colchicine ($10^{-5}$ M) | 54.6 ± 7.5 | 3.67 ± 0.35 |
| Colchicine ($10^{-5}$ M) + KT 5720 ($10^{-7}$ M) | 49.0 ± 7.1 | 3.07 ± 0.27 |
| *KT 5822* | | |
| None | 33.1 ± 6.3 | 1.83 ± 0.43 |
| KT 5822 ($10^{-7}$ M) | 35.8 ± 2.0 | 1.40 ± 0.29 |
| Colchicine ($10^{-5}$ M) | 53.0 ± 6.3 | 3.07 ± 0.43 |
| Colchicine ($10^{-5}$ M) + KT 5822 ($10^{-7}$ M) | 54.1 ± 9.6 | 2.83 ± 0.87 |

Mean of 3 experiments ± s.d.m. Cells were first preincubated in basic medium for 10 min at 37°C and then incubated with or without the agents listed in the Table for another 30 min. Then slide-coverslip preparations were made to assess locomotion.

inhibitors including staurosporine (see Table I; Meyer *et al.*, 1989), induced a dose-dependent increase in the proportion of polarised and of migrating cells.

Our findings suggest that PKC itself plays a more significant role in regulating locomotion than cyclic nucleotide-dependent protein kinases. Inhibitors (KT 5720, KT 5822) preferentially acting on this class of kinases were inactive. Modifications at the 9-hydroxy or 9-methoxy-carbonyl moieties of the K 252 molecular core (see Table I; Kase *et al.*, 1987) thus appear to drastically change the inhibitory profiles for protein kinases, and the biological effects. Protein kinase inhibitors with a relatively high selectivity for PKC, i.e. staurosporine, K 252a and CGP 41251 produced diverse effects on cell polarity and locomotion. We initially speculated that the PKC inhibitors staurosporine, K 252a or CGP 41251 *vs* activators of PKC might have opposing effects. PMA suppresses polarity and locomotion but only CGP 41251, i.e. the compound with the highest selectivity for PKC stimulated polarity and locomotion. In contrast, staurosporine and K 252a suppressed polarity and locomotion similar to PMA. Thus, only the inhibitor with the highest selectivity for PKC (i.e. CGP 41251) has effects opposite to active phorbol esters. Inhibitors with a lower selectivity (staurosporine, K 252a) have more complex inhibition profiles and their effect may not be sufficiently representative for mere PKC inhibition. They may therefore affect biological responses through several different mechanisms (including PKC) which are not yet sufficiently clear. It has been suggested, that the rapid increase in cytoskeleton-associated actin of neutrophils exposed to staurosporine is due to inhibition of an unknown staurosporine-sensitive enzyme, not identical with PKC or one of the cyclic-nucleotide-dependent kinases (Niggli & Keller, 1991). Furthermore, inhibitors of the staurosporine type seem to possess phorbol ester agonistic as well as antagonistic effects (Dlugosz & Yuspa, 1991). Interestingly, both staurosporine and PMA induce association of PKC with the neutrophil membrane (Wolf & Baggiolini, 1988) and a dendritic shape in keratinocytes (Sako *et al.*, 1988).

It is at present also rather difficult to properly understand the mechanisms involved in the action of K 252a. Several studies on K 252a also show that a biological response with a PKC inhibitor may not only depend on PKC inhibition, but may also be related to other mechanisms. K 252a seems to act on PKC as well as on PKC-like, but calcium-unresponsive protein kinases (p82 kinase and p76 kinases; the $IC_{50}$ of K 252a with regard to PKC and the p76-kinase differing by two orders of magnitude; Gschwendt *et al.*, 1989). In contrast to PKC, K 252a at concentrations of up to $5 \times 10^{-7}$ M fails to suppress p76 kinase activity, but it inhibits PKC-catalysed phosphorylation up to 50%. Furthermore, inhibition of protein phosphorylation by K 252a is still effective when the process of PMA-mediated down-regulation is completed, but K 252a does not influence PMA-induced down-regulation of PKC at all (Lindner *et al.*, 1991). Loss of K 252a-induced kinase inhibition through enzyme decay and eventual consecutive formation of catalytically active fragments (e.g., 50 kDa kinase M, which can phosphorylate phosphatidylinositol-4-phosphate; (Tusupov *et al.*, 1991) does not appear to represent a probable mechanism. Differential effects on cell motility may theoretically also depend on varying drug interactions with PKC isoforms. At least eight subspecies of PKC have been identified with differences in structure, substrate, and calcium dependence (Nishizuka, 1988; Bacher *et al.*, 1991). Interestingly, PMA treatment of intact epithelial cells increased the level of phosphorylation of major cytoskeletal compounds, i.e. cytokeratins 8/18 (Chou & Omary, 1991). An isoform (PKC epsilon)-related kinase associates with and phosphorylates cytokeratins 8 and 18 (Omary *et al.*, 1992). As the cytoskeleton controls cell shape and locomotion it will be of interest to test PKC inhibitors with regard to PKC subspecies.

Further insight into possible mechanisms may be gained by looking at the molecular structure. CGP 41 251 (Figure 1) has an aromatic ring (a benzoyl group) bound to nitrogen in close vicinity to a methyl group and a methoxy group, which after binding to PKC may alter the enzyme's interactions with a lipid environment (for review, see Bell & Burns, 1991). One may theorise that binding of an agent with a hydrophobic cluster, such as CGP 41 251, may modify the interaction with phosphatidyl-serine molecules located in cellular membranes, or modify the binding to other cell components. One mechanism may be of particular interest for understanding the effects of PKC-inhibitors on cell shape and motility. PKC does not only bind to membrane lipids, but may interact with cytoskeletal proteins in the particulate fraction and in the nuclei. Binding of PKC to two of these proteins (receptors for activated C kinase, 'RACKS') was concentration-dependent, saturable, and specific (Mochly-Rosen *et al.*, 1991). PKC binds to RACKS via a site on PKC distinct from the substrate binding site. It has been suggested that binding to RACKS may play a role in activation

(DAG)-induced translocation of PKC (Mochly-Rosen *et al.*, 1991), but RACK binding may theoretically also be altered by PKC-bound agents such as CGP 41 251.

Further studies with an extended set of alkaloid analogues with high specificity for PKC are required. Staurosporine and K 252a may not be sufficiently representative tools to study effects specifically related to PKC-inhibition.

The work was supported by the Swiss Cancer League and the Cancer League of the Canton of Glarus and the Swiss National Science Foundation. We thank Dr Thomas Meyer, CIBA GEIGY AG, Basel, for advice and for permission to use data on K 252a. The excellent technical assistance of Miss M. Kilchenmann is gratefully acknowledged.

### References

BACHER, N., ZISMAN, Y., BERENT, E. & LIVNEH, E. (1991). Isolation and characterization of PKC-L, a new member of the protein kinase C-related gene family specifically expressed in lung, skin, and heart. *Mol. Cell. Biol.*, **11**, 126–133.

BELL, R.M. & BURNS, D.J. (1991). Lipid activation of protein kinase C. *J. Biol. Chem.*, **266**, 4661–4664.

CHIARUGI, V., BRUNI, P., PASQUALI, F., MAGNELLI, L., BASI, G., RUGGIERO, M. & FARNARERO, M. (1989). Synthesis of diacylglycerols denovo is responsible for permanent activations and down-regulation of protein kinase C in transformed cells. *Biochem. Biophys. Res. Commun.*, **164**, 816–823.

CHOU, C.-F. & OMARY, B. (1991). Phorbol acetate enhances the phosphorylation of cytokeratins 8 and 18 in human colonic epithelial cells. *Fed. Eur. Biochem. Soc. Lett.*, **282**, 200–204.

DLUGOSZ, A.A. & YUSPA, S.H. (1991). Staurosporine induces protein kinase C agonist effects and maturation of normal and neoplastic mouse keratinocytes *in vitro*. *Cancer Res.*, **51**, 4677–4684.

FRIDMAN, R., LACAL, J.C., REICH, R., BONFIL, D.R. & AHN, C.H. (1990). Differential effects of phorbol ester on the *in vitro* invasiveness of malignant and non-malignant human fibroblast cells. *J. Cell. Phys.*, **142**, 55–60.

GOPALAKRISHNA, R. & BARSKY, S.H. (1988). Tumor promoter-induced membrane-bound protein kinase C regulates hematogenous metastasis. *Proc. Natl. Acad. Sci. USA*, **85**, 612–616.

GSCHWENDT, M., LEIBERSPERGER, H. & MARKS, F. (1989). Differentiative action of K 252a on protein kinase C and A. Calcium-unresponsive, phorbol ester/phospholipid-activated protein kinase. *Biochem. Biophys. Res Commun.*, **164**, 974–982.

HIDAKA, H., INAGAKI, M., KAWAMOTO, S. & SASAKI, Y. (1984). Isoquinoline sulfonamides, novel and potent inhibitors of cyclic nucleotide-dependent protein kinase and protein kinase C. *Biochemistry*, **23**, 5036–5041.

KASE, H., IWAHASHI, K., NAKANISHI, S., MATSUDA, Y., YAMADA, K., TAKAHASHI, M., MURAKATA, C., SATO, A. & KANEKO, M. (1987). K-252 compounds, novel and potent inhibitors of protein kinase C and cyclic nucleotide-dependent protein kinases. *Biochem. Biophys. Res. Commun.*, **142**, 436–440.

KELLER, H.U. (1983). Motility, cell shape and locomotion of neutrophil granulocytes. *Cell Motility*, **3**, 47–60.

KELLER, H.U., ZIMMERMANN, A. & COTTIER, H. (1985). Phorbol myristate acetate (PMA) suppresses polarization and locomotion and alters F-actin content of Walker carcinosarcoma cells. *Int. J. Cancer*, **36**, 495–501.

KELLER, H.U. & ZIMMERMANN, A. (1986). Shape changes and chemokinesis of Walker 256 carcinosarcoma cells in response to colchicine, vinblastine, nocodazole and taxol. *Invasion Metastasis*, **6**, 33–43.

KELLER, H.U., ZIMMERMANN, A. & NIGGLI, V. (1989). Diacylglycerols and the protein kinase inhibitor H-7 suppress cell polarity and locomotion of Walker 256 carcinosarcoma cells. *Int. J. Cancer*, **44**, 934–939.

LINDNER, D., GSCHWENDT, M. & MARKS, F. (1991). Down-regulation of protein kinase C in Swiss 3T3 fibroblasts is independent of its phosphorylating activity. *Biochem. Biophys. Res. Commun.*, **176**, 1227–1231.

MEYER, T., REGENASS, U., FABBRO, D., ALTERI, E., RÖSEL, J., MÜLLER, M., CARAVATTI, G. & MATTER, A. (1989). A derivative of staurosporine (CGP 41 251) shows selectivity for protein kinase C inhibition and *in vitro* anti-proliferative as well as *in vivo* anti-tumor activity. *Int. J. Cancer*, **43**, 851–856.

MOCHLY-ROSEN, D., KHANER, H. & LOPEZ, J. (1991). Identification of intracellular receptor proteins for activated protein kinase C. *Proc. Natl. Acad. Sci. USA*, **88**, 3997–4000.

NIGGLI, V. & KELLER, H.U. (1991). On the role of protein kinases in regulating neutrophil actin association with the cytoskeleton. *J. Biol. Chem.*, **266**, 7927–7932.

NISHIZUKA, Y. (1988). The molecular heterogeneity of protein kinase C and its implications for cellular regulation. *Nature*, **334**, 661–665.

OMARY, M.B., BAXTER, G.T., CHOU, C.F., RIOPEL, C.L., LIN, W.Y. & STRULOVICI, B. (1992). $PKC_E$-related kinase associates with and phosphorylates cytokeratin 8 and 18. *J. Cell Biol.*, **117**, 583–593.

PREISS, J., LOOMIS, C.R., BISHOP, W.R., STEIN, R., NIEDEL, J.E. & BELL, R.M. (1986). Quantitative measurement of SN-1,2-diacylglycerols present in platelets, hepatocytes and ras- and sis-transformed normal rat kidney cells. *J. Biol. Chem.*, **261**, 8597–8600.

ROOS, F.J., ZIMMERMANN, A. & KELLER, H.U. (1987). Effect of phorbol myristate acetate and the chemotactic peptide fNLPNTL on shape and movement of human neutrophils. *J. Cell Sci.*, **88**, 399–406.

RÜEGG, U.T. & BURGESS, G.M. (1989). Staurosporine, K252 and UCN-01: potent but nonspecific inhibitors of protein kinases. *TIBS*, **10**, 218–220.

SAKO, T., TAUBER, A.I., JENG, A.Y., YUSPA, S.H & BLUMBERG, P.M. (1988). Contrasting actions of staurosporine, a protein kinase C inhibitor, on human neutrophils and primary mouse epidermal cells. *Cancer Res.*, **48**, 4646–4650.

TAKENAGA, K. & TAKAHASHI, K. (1986). Effects of 12-0-tetradecanoyl-phorbol-13-acetate on adhesiveness and lung-colonizing ability of Lewis lung carcinoma cells. *Cancer Res.*, **40**, 375–380.

TUSUPOV, O.K., SEVERIN, S.E. & SHVETS, V.I. (1991). Proteolytic fragment of protein kinase C (kinase M) phosphorylates in vitro phosphatidylinositol-4-phosphate. *Biochem. Biophys. Res. Commun.*, **176**, 1007–1013.

WEYMAN, C.N. (1988). Partial down-regulation of protein kinase C in C3H10T mouse fibroblasts transfected with the human Ha-ras oncogene. *Cancer Res.*, **48**, 6535–6541.

WOLF, M. & BAGGIOLINI, M. (1988). The protein kinase inhibitor staurosporine, like phorbol esters, induces the association of protein kinase C with membranes. *Biochem. Biophys. Res. Commun.*, **154**, 1273–1279.

ZIMMERMANN, A. & KELLER, H.U. (1987). Locomotion of tumor cells as an element of invasion and metastasis. *Biomed. Pharmacol.*, **41**, 337–344.

ZIMMERMANN, A., GEHR, P. & KELLER, H.U. (1988). Diacylglycerol-induced shape changes, movements and altered F-actin distribution in human neutrophils. *J. Cell Sci.*, **90**, 657–666.